# The PTP1B selective inhibitor MSI-1436 mitigates Tunicamycin-induced ER stress in human hepatocarcinoma cell line through XBP1 splicing modulation

**DOI:** 10.1371/journal.pone.0278566

**Published:** 2023-01-17

**Authors:** Lynda Bourebaba, Sai Santosh Babu Komakula, Christine Weiss, Nabil Adrar, Krzysztof Marycz

**Affiliations:** 1 Faculty of Biology and Animal Science, Department of Experimental Biology, Wrocław University of Environmental and Life Sciences, Wrocław, Poland; 2 International Institute of Translational Medicine, Jesionowa, Wisznia Mała, Poland; 3 PferdePraxis Dr. Med. Vet. Daniel Weiss, Freienbach, Switzerland; 4 Department of Medicine and Epidemiology, UC Davis School of Veterinary Medicine, Davis, CA, United States of America; Augusta University, UNITED STATES

## Abstract

Protein tyrosine phosphatase PTP1B is considered as a key metabolic enzyme that has been reported to be associated with insulin resistance onset, and underlying cellular metabolic malfunctions, including ER stress and mitochondrial failure. In this study, effects of selective PTP1B inhibition using MSI-1436 on cellular apoptosis, oxidative stress, mitochondrial dysfunction and ER stress have been assessed using an *in vitro* model of Tunicamycin induced ER stress in HepG2 cell line. Inhibition of PTP1B using MSI-1436 significantly increased cell viability and reduced the number of apoptotic cells as well as the expression of key apoptosis initiators and effectors. MSI-1436 further mitigated ER stress, by downregulating the expression of IRE1, ATF6 and PERK transcripts, all being key ER stress sensors. Interestingly, MSI-1436 inhibited the XBP1 splicing, and thus its UPR-associated transcriptional activity. PTP1B inhibition further enabled to restore proper mitochondrial biogenesis, by improving transmembrane potential, and diminishing intracellular ROS while restoring of endogenous antioxidant enzymes genes expression. PTP1B inhibition using MSI-1436 could improve cellular apoptosis and metabolic integrity through the mitigation of ER and mitochondrial stress signalling pathways, and excessive ROS accumulation. This strategy may be useful for the treatment of metabolic disorders including IR, NAFLD and diabetes.

## 1 Introduction

Liver plays an essential role in regulating heath and disease by controlling energy homeostasis and eliminating potential toxic metabolites [1–3]. Due to complex functionality of liver and enterohepatic circulation hepatocytes constantly exposed to variety of dietary and Gut microbiome derived metabolites, some of these metabolites can be hazardous and could cause hepatocyte apoptosis [4–6]. On the other hand, excess caloric intake also results in obesity and hepatic steatosis [7,8]. Hepatic steatosis can progress into non-alcoholic steatohepatitis (NASH), which can further lead to deadly HCC development. Liver is an active organ involved in several secretory factors including albumin and lipoproteins. Liver ER plays major role in controlling whole body energy homeostasis, several important steps including packaging of lipoproteins and unsaturation of fatty acids takes place in ER [9,10]. Moreover, ER membrane maintains tight connection with mitochondria outer membrane forming mitochondria-associated membranes (MAM), which regulates mitochondrial lipid intake, calcium homeostasis and autophagy [11–13]. Several studies have demonstrated that, hepatic steatosis leads to increased ER stress and mitochondrial dysfunction [14,15]. Recently it’s also been shown that ER stress can promote NASH development via IRE1a-casp2 axis. IRE1a regulates Casp2 expression, which cleaves Site-1 protease (S1P) and activates SREBP1 to induce lipid synthesis [16,17]. Chronic ER stress in hepatocytes leads to initiation of apoptotic signalling cascade and hepatocyte death [18–21]. CHOP plays major role in ER stress induced apoptosis by promoting the expression of pro-apoptotic proteins death receptor 5 (DR5), B-cell lymphoma 2 like protein 11 (BIM), growth arrest and DNA damage-inducible protein (GADD34) and tribbles homolog 3 (TRB3). CHOP also shown to downregulate anti-apoptotic B-cell lymphoma 2 (BCL2) protein [21,22]. Moreover ER stress induced NASH model displayed increased CHOP expression in the liver, and also demonstrated that mice with excessive ER stress have more propensity to develop NASH and HCC [23,24]. Hepatocyte apoptosis is also strongly associated with the progression of HCC from NASH, mice with increased hepatocyte apoptosis are more prone to develop HCC from NASH [25]. Hepatocytes undergoing apoptosis release damage associated molecular patterns (DAMPs), which can accelerate the progression of NASH and increase the chance of HCC development by initiating the inflammatory signalling [26,27]. Thus, reducing hepatocyte ER stress and apoptosis could be beneficial in NASH, ALD and cholestatic liver disease [28]. In this regard, potentially safe small molecules which can protect hepatocytes from ER stress and subsequent apoptosis are needed to attenuate the progression of liver diseases.

ER stress in obese patients relates to leptin and insulin resistance not only in peripheral tissues but also in central tissue, as leptin and insulin are important anorexigenic hormones acting on the hypothalamus, which exhibited elevated expression of ER stress markers such as PERK and IRE1 in the course of obesity and associated pathologies [29,30]. Although the molecular mechanism underlying leptin and insulin resistance in obesity is still incompletely understood, protein-tyrosine phosphatase (PTP) 1B has been demonstrated as a major negative regulator of insulin and leptin sensitivity. Protein tyrosine phosphatases (PTPases) are Redox sensitive enzymes, which are known to negatively regulate insulin signal pathways, as they are able to dephosphorylate phosphotyrosine residues of the insulin receptor (*Insr*) and *Irs-1*. In physiological stat, insulin induces ROS-mediated oxidation of critical amino acids moieties of the PTPase catalytic domain, triggering its inactivation, which subsequently enhances signal transduction events downstream of the *Insr* [31,32].

PTP1B is a protein naturally attached to the endoplasmic reticulum via its C-terminal targeting sequence, which has been proposed as a critical molecular link associating ER stress to insulin resistance and obesity onset. Previous study demonstrated that PTP1B overactivity correlates with elevated ER stress in the course of obesity, and that diminished ER stress and related effectors resulted in the downregulation of obesity-associated elevated PTP1B level. On the other hand, specific PTP1B deletion has been found to attenuate ER-stress-mediated JNK activation, X-box binding protein 1 (XBP-1) splicing and apoptosis, while alleviating high-fat diet-induced ER stress response in mice [33].

In this context, PTP1B has been strongly considered as potentially promising drug targets for the prevention and treatment of insulin resistance and T2DM and associated cellular malfunctions including ER stress [34]. Several synthetic and natural molecules, as well as antisense oligonucleotides have already demonstrated their effectiveness in reversibly inhibiting PTPs, these substances were mainly grouped into four main classes including including: difluoromethylene phosphonates, 2-carbomethoxybenzoic acids, 2-oxalylaminobenzoic acids and lipophilic compounds. Thereby, the development of inhibitors of PTP enzymes has offered new perspectives in understanding the design and function of phosphatase-based drugs, for the prevention and treatment of insulin resistance and underlying metabolic conditions [35]. Trodusquemine or MSI-1436 is an aminosterol isolated from tissues of the dogfish shark *Squalas acanthias* which selectively inhibits the PTB1B enzyme in a non-competitive manner, thereby represents a promising drug candidate for the management of PTP1B overactivation associated pathologies [36]. Recently, our research group showed that MSI-1436 improves EMS adipose derived progenitor stem cells in the course of adipogenic differentiation through the modulation of ER stress, apoptosis, and oxidative stress via the inhibition of PTP1B activity [37]. Another study in diet-induced obese mice showed that MSI-1436 suppressed appetite, reduced body weight, improved plasma insulin and leptin levels through selective inhibition of PTP1B [38]. Thus, in the present investigation, we sought to identify the effects of PTP1B inhibition using its selective inhibitor MSI-1436 on ER stress signalling pathways in HepG2 cells exposed to tunicamycin, and whether these changes could reduce apoptosis and improve mitochondrial biogenesis.

## 2 Materials and methods

### 2.1 Cell culture and treatments

Human hepatocarcinoma HepG2 cell line (ATCC® HB-8065™) was obtained from the *American Type Culture Collection* (Manassas, VA, USA). Cells were cultured in low-glucose *Dulbecco’s modified Eagle’s medium* (DMEM, Gibco Carlsbad, CA, USA) supplemented with 10% heat inactivated foetal bovine serum (FBS) (Gibco Carlsbad, CA, USA). Cells were cultured at 37°C in a humidified chamber with 5% CO_2,_ then passaged every 3 days using a Trypsin/EDTA solution (Gibco Carlsbad, CA, USA). Tunicamycin (Cayman Chemical, Cat. No: 11445, USA) was used to induce endoplasmic reticulum (ER) stress in cultured hepatocytes. Four testing, cell culture groups were established, (1): healthy (HE) cells, (2): ER stressed cells by 5 μM of tunicamycin for 24 hours (ERS), (3) Pre-treatment with 1μM MSI-1436 for 1 hour followed by cotreatment with 1μM MSI-1436 and 5μM tunicamycin (ERS+MSI-1436_1μM), and (4): Pre-treatment with 2uM MSI-1436 for 1 hour followed by cotreatment with 2μM MSI-1436 and 5μM tunicamycin (ERS+MSI-1436_2μM).

### 2.2 Cell proliferation determination

After 24h treatment with MSI-1436 at different concentrations and ER stress induction using tunicamycin, the proliferation rate of HepG2 cells was evaluated using a 10% resazurin-based dye-TOX-8 (Sigma Aldrich, Poznań, Poland) solution according to the manufacturer’s protocols. In brief, the culture media were removed following each previously described treatments, and then replaced with the fresh culture medium containing 10% resazurin dye solution and incubate at 37°C for 2 hours. Absorbance was measured at a wavelength of 600 nm for resazurin and at the 690 nm reference wavelength using a microplate reader (BMG Labtech, Germany).

### 2.3 Flow cytometric analysis of cell viability and apoptosis

Cell viability and Apoptosis initiation was determined using the MUSE™ Annexin V & Dead Cell Kit (Merck Millipore, Darmstadt, Germany) according to the manufacturer’s instructions. Briefly, after treatment with MSI-1436 and exposure to tunicamycin, all treated and untreated Hep-G2 cells were collected by trypsinization and suspended in HBSS containing 1% FBS. Then, cells were stained with the Annexin V & Dead Cell Kit for 20 minutes at room temperature and analyzed using the Muse cell analyzer (Merck Millipore, Darmstadt, Germany). The apoptotic ratio was calculated by the identification of four populations: (i) non-apoptotic cells, not undergoing detectable apoptosis: Annexin V (-) and 7-AAD (-); (ii) early apoptotic cells, Annexin V (+) and 7-AAD (-); (iii) late apoptotic cells, Annexin V (+) and 7-AAD (+); (iv) cells that died through non-apoptotic pathway: Annexin V (-) and 7-AAD (+).

### 2.4 Intracellular ROS assessment

Intracellular reactive oxygen species (ROS) were determined using the Muse® Oxidative Stress Kit based on dihydroethidium (DHE). All procedures were performed according to the protocols provided by the manufacturer. Cells were collected using Trypsin–EDTA 1X in HBSS following the above-described treatments, washed with HBSS and mixed with the Muse Oxidative Stress Reagent working solution prepared in 1X Assay Buffer. After 30 min incubation at 37°C, samples were subjected to Muse cell analyzer (Merck, Darmstadt, Germany), and two cell populations were detected: Live ROS cells (-) and ROS (+) cells exhibiting high content of ROS.

### 2.5 Mitochondrial transmembrane potential (MMP) analysis

Depolarization of the mitochondrial inner membrane (ΔΨ) was evaluated using Muse™ MitoPotential Kit from Merck Millipore according to supplier’s recommendations. HepG2 cultured under the same previously detailed experimental conditions were collected using Trypsin–EDTA 1X, washed with HBSS and mixed with the MitoPotential working solution prepared in 1X Assay Buffer (1:1000), and incubated during 20 min at 37°C. After incubation time, Muse Mito-Potential 7-AAD reagent was added to the cells suspension and further incubated for 5 min at room temperature in the dark. Cells were analyzed using the Muse™ Cell Analyzer equipped with Muse™ Software. The total percentage of cells with depolarized mitochondrial membrane was then acquired.

### 2.6 Mitochondrial network staining

Mitochondrial network has been visualized using a confocal microscope (Observer Z1 Confocal Spinning Disc V.2 Zeiss with live imaging chamber). In brief, cells seeded onto glass coverslips were labelled with the MitoRed fluorescent tag (Sigma Aldrich, Poznań, Poland) prepared in fresh culture medium (1:1000) following each related treatment for 30 min at 37°C, and subsequently fixed in 4% PFA for 40 min at room temperature. Cell nuclei were counterstained with 4′,6-diamidino-2-phenylindole (DAPI), using the ProLong™ Diamond Antifade Mountant with DAPI (Invitrogen™, Poland). Photomicrographs were captured as z-stacks having a z-interval of 15, 20, or 25 μm between two consecutive optical slices at a digital size of 512 × 512 pixels using a Canon PowerShot camera. Obtained photomicrographs were merged using ImageJ software (Bethesda, MD, USA). Mitochondria morphology analysis was performed on six individual cells per experimental group using the MicroP software (ver. 1.1.11b, Biomedical Image Informatics Lab, Taipei City, Taiwan (R.O.C.) Institute of Biomedical Informatics, National Yang Ming Chiao Tung University) powered by MATLAB (version R2010b, TheMathWorks, Natick, MA, USA) [39].

### 2.7 Real-Time Reverse Transcription Polymerase Chain Reaction Analysis (Rt-qPCR)

To examine the ability of MSI-1436 to prevent ER stress and its consequent impact on cell function, the expression levels of transcripts involved in apoptosis, ER stress, as well as mitochondrial dynamics was assessed by RT-qPCR. Total RNA was isolated from cells of each tested group using TRIzol reagent (Sigma Aldrich, Poznań, Poland), according to the manufacturer’s instructions, followed by concentration and purity measurements using a nanospectrophotometer (WPA, Biowave II, Germany). Genomic DNA (gDNA) digestion and cDNA synthesis were performed by reverse transcription reaction with oligo(dT) primers using a Tetro cDNA Strand cDNA Synthesis Kit (Bioline, London, UK) in a T100 Thermal Cycler (Bio-Rad, Hercules, CA, USA) according to the provided kit instructions. Quantification of relative gene expression was performed in a CFX Connect™ Real-Time PCR Detection System (Bio-Rad) using SensiFAST SYBR Green Kit (Bioline, London, UK) for the detection of targeted mRNA expression. A total of 150 ng of cDNA from each sample was amplified with each combination of forward and reverse primers (listed in Table 1), and SYBR-Green Master Mix, in a total volume of 10 μl. The thermal cycling conditions were as follows: initial denaturation at 95°C for 2 min, 40 cycles at denaturation at 95°C for 15 s followed by annealing for 15 s and finally elongation at 72°C for 15 s. A dissociation curve analysis was performed at the end of each run. The 2^ − ΔΔCt^ method was used to calculate relative quantification of mRNA content; Ct values were normalized to the expression of the house-keeping genes glyceraldehyde-3-phosphate dehydrogenase (GAPDH) and β-actin (ACTB). XBP1 amplification product was further run on a 2% agarose gel to visualize the spliced and unspliced XBP1 sequences (un-spliced XBP1: 281bp, spliced: 255bp). Bands were quantified using Image Studio Lite software (LI-COR Biosciences, USA).

**Table 1:** Sequences of primers (Sigma Aldrich, Poznań, Poland) used in qPCR.

Gene	Primer	Sequence 5’–3’	Amplicon length (bp)	Accession No.
*ATF6*	F: R:	ACCTCCTTGTCAGCCCCTAA CACTCCCTGAGTTCCTGCTG	150	NM_007348.4
*IRE1*	F: R:	CGGCCTCGGGATTTTTGGA AGAAAGGCAGGCTCTTCCAC	110	NM_001433.5
*BiP*	F: R:	CGGCCTCGGGATTTTTGGA AGAAAGGCAGGCTCTTCCAC	177	NM_001433.5
*CHOP*	F: R:	TAAAGATGAGCGGGTGGCAG GGATAATGGGGAGTGGCTGG	103	NM_001195053.1
*Casp3*	F: R:	CTCTGGTTTTCGGTGGGTGT CTTCCATGTATGATCTTTGGTTCC	136	NM_004346.4
*Casp6*	F: R:	TCATGAGAGGTTCTTTTGGCAC CACACACAAAGCAATCGGCA	197	NM_001226.4
*Casp9*	F: R:	CAGGCCCCATATGATCGAGG CTGGCCTGTGTCCTCTAAGC	142	NM_032996.3
*CAT*	F: R:	ACCAAGGTTTGGCCTCACAA TTGGGTCAAAGGCCAACTGT	112	XM_014851065.1
*Fis1*	F: R:	TGGTGCGGAGCAAGTACAAT TGCCCACGAGTCCATCTTTC	132	NM_016068.3
*GPx*	F: R:	TCCGGGACTACACCCAGATG TCTTGGCGTTCTCCTGATGC	108	NM_000581.4
*Mfn1*	F: R:	GTTGCCGGGTGATAGTTGGA TGCCACCTTCATGTGTCTCC	146	NM_033540.3
*Mfn2*	F: R:	AATCTGAGGCGACTGGTGAC GGACATTGCGCTTCACCTTC	126	XM_024451299.1
*p21*	F: R:	AGAAGAGGCTGGTGGCTATTT CCCGCCATTAGCGCATCAC	169	NM_001220777.1
*p65*	F: R:	CTGTTCCCCCTCATCTTCCC GTATCTGTGCTCCTCTCGCC	156	NM_001243985.2
*Perk*	F: R:	TGCTCCCACCTCAGCGAC TTTCAGGATCCAAGGCAGCA	124	NM_004836.6
*Pink1*	F: R:	GCTTGGGACCTCTCTTGGAT CGAAGCCATCTTGAACACAA	142	NM_032409.3
*Parkin*	F: R:	GTGCAGAGACCGTGGAGAAA GCTGCACTGTACCCTGAGTT	294	NM_013987.3
*Sod1 (cu/zn sod)*	F: R:	CATTCCATCATTGGCCGCAC GAGCGATCCCAATCACACCA	130	NW_001867397.1
*Sod2 (cu/zn sod)*	F: R:	GGACAAACCTGAGCCCCAAT TTGGACACCAGCCGATACAG	125	NW_001867408.1
*GAPDH*	F: R:	GTCAGTGGTGGACCTGACCT CACCACCCTGTTGCTGTAGC	256	NM_001289746.1

***Atf6*:** Activating transcription factor 6; ***IRE1*:** Inositol-requiring enzyme; ***BiP*:** Binding immunoglobulin protein; ***CHOP*:** C/EBP homologous protein; ***Casp3*:** Caspase 3; ***Casp6*:** Caspase 6; ***Casp9*:** Caspase 9; ***CAT*:** Catalase; ***Fis1*:** Mitochondrial fission 1 molecule; ***GPx*:** Glutathione Peroxidase; ***Mfn1*:** Mitofusin 1; ***Mfn2*:** Mitofusin 2; ***p21*:** Cyclin-dependent kinase inhibitor 1; ***p65*:** RELA proto-oncogene, NF-kB subunit (RELA), transcript variant 4; ***Perk*:** Protein Kinase RNA-like Endoplasmic Reticulum Kinase; ***Pink1*:** PTEN-induced putative kinase 1; ***Parkin*:** RBR E3 ubiquitin protein ligase; ***Sod1 (Cu/Zn SOD)*:** Copper-zinc-dependant superoxide dismutase; ***Sod2 (Mn SOD)*:** Manganese-dependent superoxide dismutase; ***GADPH*:** Glyceraldehyde-3-phosphate dehydrogenase.

### 2.8 Statistical analysis

All experiments were performed in triplicate. Data are presented as mean ± standard deviation (SD). Differences between means were determined by one-way analysis of variance (ANOVA) then, differences among experimental groups were compared using the *Tukey’s* post-test (*GraphPad Prism 9*, San Diego, CA). Results were considered statistically significant when *p < 0*.*05* (marked with asterisk*), those with *p < 0*.*01*, *p < 0*.*001* and *p < 0*.*0001* are respectively marked with **, *** and ****.

## 3 Results

### 3.1 MSI-1436 prevents apoptosis in TM-induced ER stress in HepG2 cells

**Fig 1:**
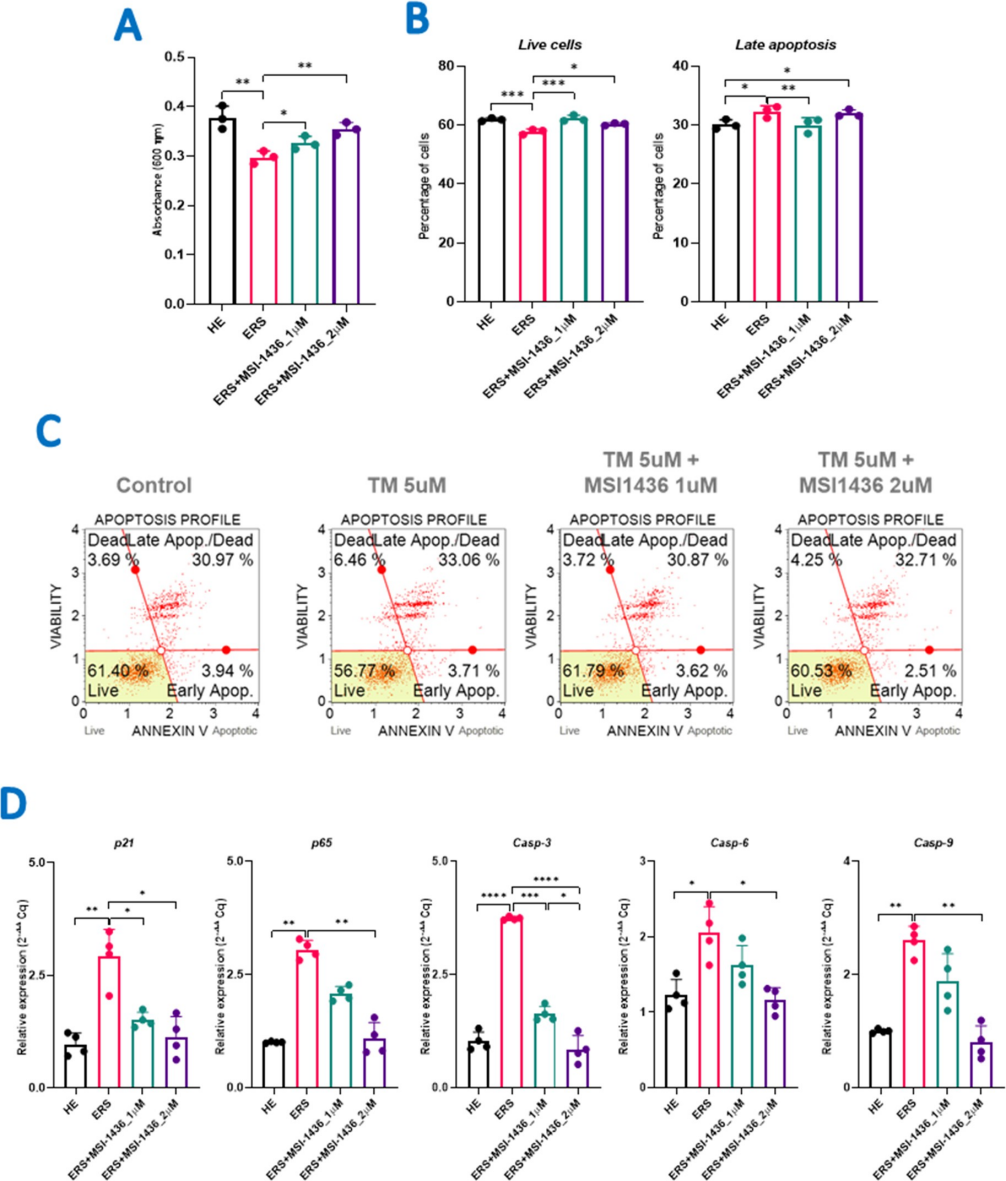
Evaluation of cell viability and apoptosis in MIS-1436 preconditioned or non-treated HepG2 cells following TM-induced ER stress. (**A**) Proliferative rate of treated and untreated HepG2 cells assessed using the Tox8 assay. **(B)** Bar charts depicting the percentage of total live and apoptotic cells. **(C)** Annexin V & Dead Cells representative plots among three replicates of each determination. **(D)** Relative gene expression of key apoptosis associated regulators and effectors. The results are expressed as the mean of 3 different experiments ± SD. Asterisks refer to a significant difference between two groups, where: p < 0.05 (*); p < 0.01(**); p < 0.001(***); p < 0.0001 (****). HE: Healthy untreated HepG2 cells; ERS: Endoplasmic reticulum-stressed cells; ERS+MSI: Groups pretreated with MSI-1436 compound before inducing ERS.

In order to evaluate the effects of PTP1B inhibition in the course of tunicamycin (TM)-induced apoptosis, treated HepG2 cells were tested for proliferation, viability and apoptosis changes. As shown in Fig 1A., Tunicamycin application significantly reduced the proliferative rate of HepG2 when compared to healthy group of cells (*p<0*.*01*), while pre-treatment with MSI-1436 enabled to minimize the TM cytotoxic effects and thus enhance cell proliferation (p<0.05). Consistently, the developed Endoplasmic reticulum stress (ERS) had significantly affected HepG2 cell viability (*p < 0*.*001*), resulting in a substantial increase in the total number of apoptotic cells, in comparison to healthy control group (*p<0*.*05*), and which was efficiently prevented by MSI-1436 pre-treatment (Fig 1B). The further investigation on possible mechanisms by which MSI-1436 compound could protect HepG2 cells from apoptosis and death led to the following conclusions (Fig 1D): ERS had significantly induced the expression of the apoptosis regulators *p21* (*p < 0*.*01*); *p65* (*p < 0*.*01*) and the caspase mediated apoptosis effectors: *Casp-3* (*p < 0*.*0001*); *Casp-6* (*p < 0*.*05*); and *Casp-9* (*p < 0*.*01*). MSI-1436 pre-treatment and consequent PTP1B inhibition enabled to regulate the expression of the aforementioned transcripts in a dose dependent manner.

### 3.2 MSI-1436 mitigates tunicamycin-induced ER stress in HepG2 cells through the modulation of XBP1 expression

**Fig 2:**
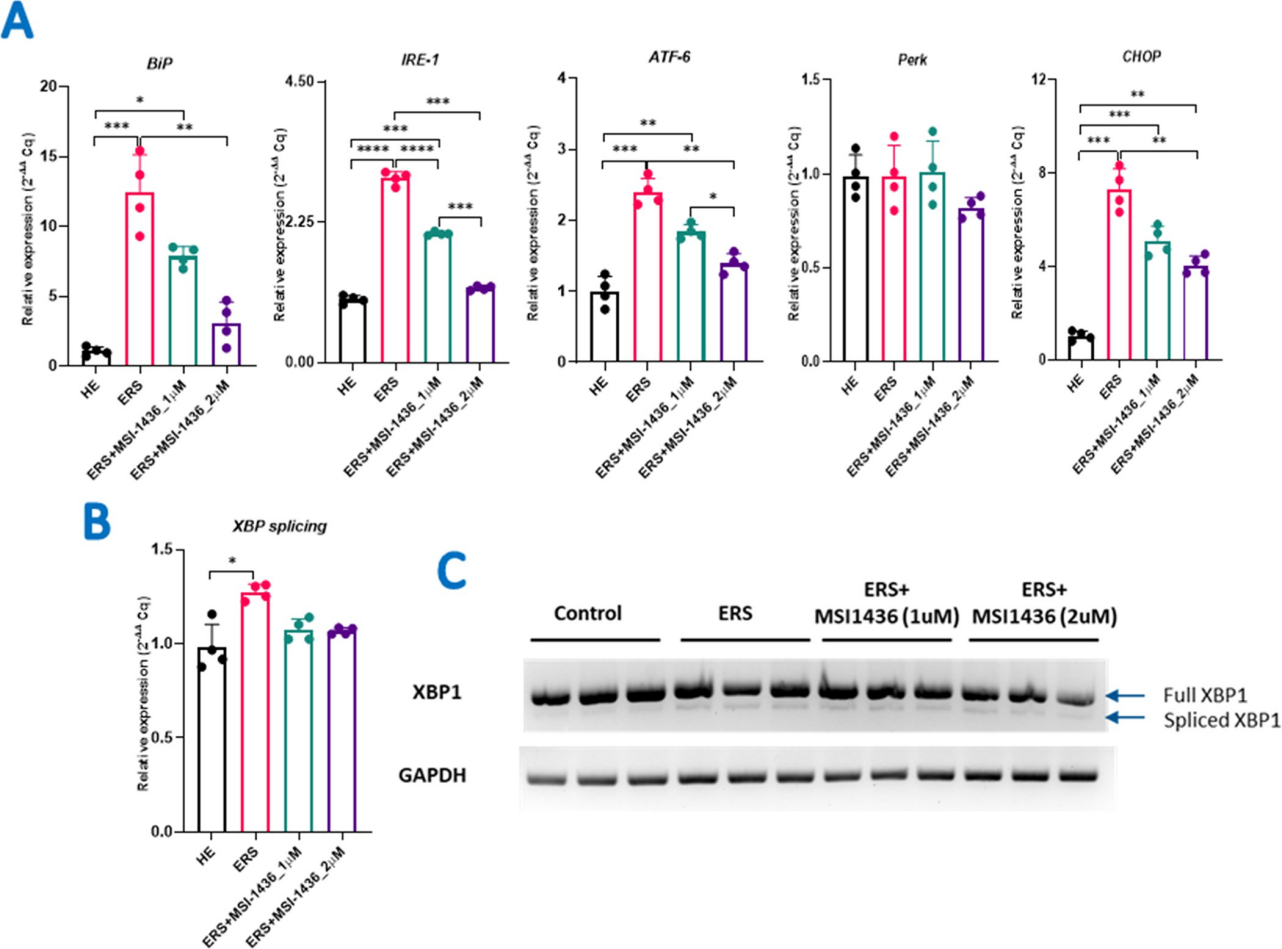
Protective effect of MSI-1436 on Tunicamycin-induced ER-stress in HepG2 cells. (**A**) Representative bar charts of the relative gene expression of ER-stress markers. (**B**) Histogram showing the relative expression of spliced XBP1 in comparison to the full-length expression. (**C**) The gel electrophoresis pattern of the spliced and unspliced amplicons as studied by RT-PCR. The results are expressed as the mean of 3 different experiments ± SD. Asterisks refer to a significant difference between two groups, where: p < 0.05 (*); p < 0.01(**); p < 0.001(***); p < 0.0001 (****). HE: Healthy untreated HepG2 cells; ERS: Endoplasmic reticulum-stressed cells; ERS+MSI: Groups pretreated with MSI-1436 compound before inducing ERS.

The relative gene expression analysis of Tunicamycin-treated HepG2 cells revealed an overactivation of ER stress-related effectors (S1 Table) as shown by the significant upregulation of: *Ire1* (*p < 0*.*0001*); *Atf6* (*p < 0*.*001*)*; Chop* (*p < 0*.*001*); and *BiP* (*p < 0*.*001*), which indicted the induction of ERS in these cells (Fig 2A). MSI-1436 supplementation resulted in a substantial downregulation of aforementioned transcripts levels; with a culminative effect observed at the highest concentration i.e., 2μM. Despite the 1μM dose was less effective, it had mitigated ERS at different levels of significance regarding each tested ER stress mediators. Changes in *Perk* expression were not significant (*p > 0*.*05*). Moreover, the quantitative estimation of spliced *Xbp1* (*sXbp1*) mRNA indicated higher relative intensity (*p* < 0.05) in ERS HepG2 cells compared to healthy cells (Fig 2B). The pre-treatment with MSI-1436 significantly reduced the *Xbp1* splicing and thus its downstream activation (S1 Fig).

### 3.3 MSI-1436 ameliorates Mitochondrial dynamics in ER stressed HepG2 cells

**Fig 3:**
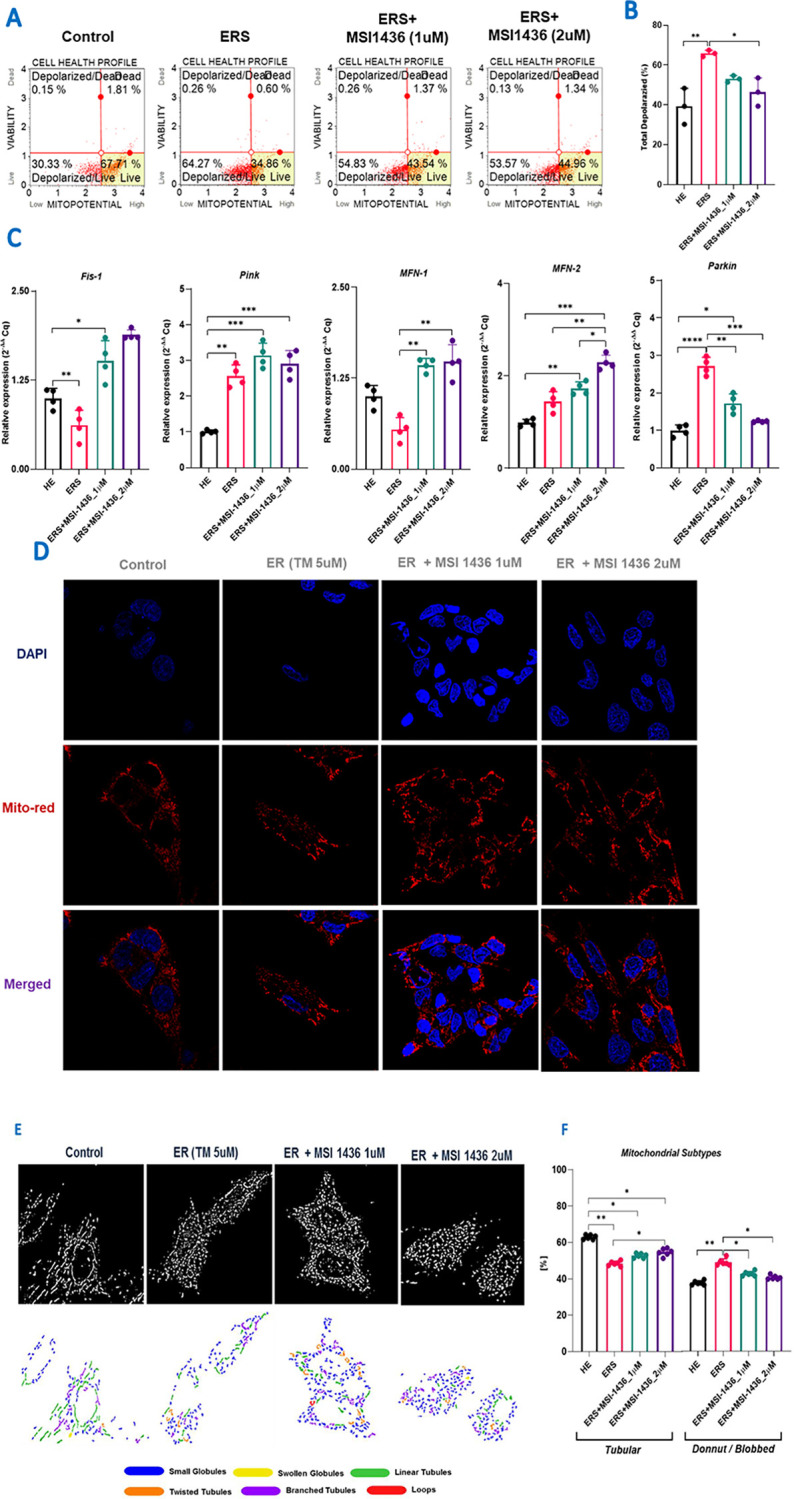
MSI-1436 improves mitochondrial dynamics in TM-challenged HepG2 cells. (**A**) Flow cytometry analysis on mitochondrial membrane potential profile. **(B)** Histogram representing the percentages of depolarized cells. **(C)** Histograms summarizing the relative expression of genes implicated in mitochondrial dynamics. (**D**) Confocal photomicrographs for Mitochondrial network stained with fluorescent Red MitoTracker. **(E)** Mitochondrial Network morphology analysis. **(F)** Bar charts depicting the percentage of tubular (including Linear, Twisted and Branched Tubules) and blobbed (including small/swollen globules and loops) mitochondria in each group of cells. The results are expressed as the mean of 3 different experiments ± SD. Asterisks refer to a significant difference between two groups, where: p < 0.05 (*); p < 0.01 (**); p < 0.001 (***); p < 0.0001 (****). HE: Healthy untreated HepG2 cells; ERS: Endoplasmic reticulum-stressed cells; ERS+MSI: Groups pretreated with MSI-1436 compound before inducing ERS.

Acute and persistent ER stress is known to ultimately lead to profound mitochondrial failure. In order to elucidate whether PTP1B inhibition may reverse the ER-stress mediated mitochondrial alteration, cells were subjected to flow cytometry and RT-qPCR analysis as well as confocal microscopy. Exposure of HepG2 cells to Tunicamycin led to a significant loss in mitochondrial transmembrane potential as evidenced by the increase in the number of cells exhibiting depolarized mitochondria (Fig 3B), by contrast to healthy control group (p<0.001). Moreover, ER stressed cells presented increased expression of mitochondrial fission mediator *Fis1* and mitophagy factors including Pink and Parkin (Fig 1C). Fusion associated marker *MFN1* appeared to be markedly downregulated in the same group of cells (p<0.01). MSI-1436 pre-treatment resulted in potent protective effect against TM induced mitochondrial dysfunction, through the maintenance of mitochondrial transmembrane potential, and regulation of fusion, fission and mitophagy related transcripts, as shown in Fig 3. Interestingly, TM application triggered to an increase in MFN2 gene expression, which has been further increased in groups pre-treated with PTP1B inhibitor (Fig 3C).

The changes in mitochondrial network morphology have also been investigated using the MitoRed fluorescent staining. Obtained photomicrographs showed that TM induced mitochondrial fission, as evidenced by the abundance of fragmented mitochondria and the increased percentage of small blobbed globules (Fig 3F), by contrast to MSI-1436 pre-treated cells, which displayed enhanced mitochondrial fusion, and more visible tubular morphology of mitochondria (Fig 3D and 3F), indicating that PTP1B inhibition may provide support in maintaining mitochondrial network integrity.

### 3.4 MSI-1436 inhibitor reduces oxidative stress in TM stressed HepG2 cells

**Fig 4:**
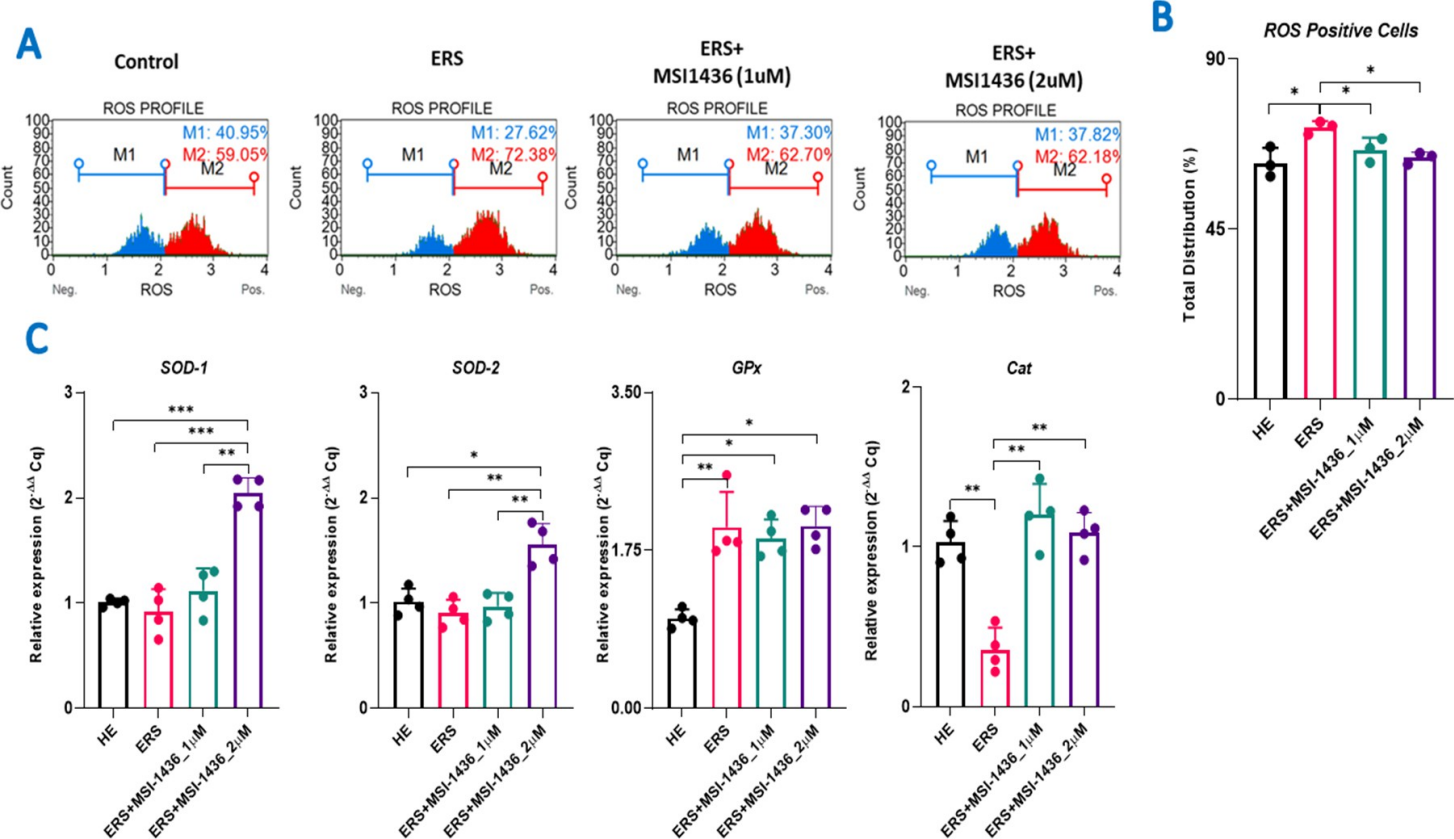
MSI-1436 compound alleviates oxidative stress in TM-treated HepG2 cells. (**A**) Representative plots of cells stained with DHE evaluated using a flow cytometer. (**B**) Bar charts summarizing the total intracellular ROS positive cells. (**C**) Representative bar charts of endogenous antioxidant enzymes-related transcripts. The results are expressed as the mean of 3 different experiments ± SD. Asterisks refer to a significant difference between two groups, where: p < 0.05 (*); p < 0.01(**); p < 0.001 (***); p < 0.0001 (****). HE: Healthy untreated HepG2 cells; ERS: Endoplasmic reticulum-stressed cells; ERS+MSI: Groups pretreated with MSI-1436 compound before inducing ERS.

Mitochondrial metabolism disruption leads to overproduction and accumulation of toxic ROS that trigger to oxidative stress occurrence and underlying cellular alterations. Oxidative status of treated and untreated HepG2 cells has been evaluated by measuring ROS levels and expression of key endogenous antioxidant enzymes. As demonstrated in Fig 4B, TM-challenged cells were characterized by elevated level of ROS positive cells, in opposition to healthy unchallenged cells (p<0.05), underlying the initiation of an oxidative stress. Cells preconditioned with MSI-1436 exhibited by contrast normalized levels of ROS positive cells, that appeared to be comparable to healthy cells, referring to a probable antioxidant effect stemming from PTP1B inhibition. In point of fact, gene expression analysis of various endogenous antioxidant enzymes, namely *Sod1*; *Sod2*; *Cat* and *GPx*; revealed that MSI-1436 compound restored the expression levels of *Sod1*, *Sod2* and *Cat*, while ER stressed and untreated cells displayed altered expression patterns for the same transcripts, indicating an imbalanced endogenous antioxidant system (Fig 4C). In contrary, GPx mRNA was found to be upregulated in ERS cells *(p < 0*.*01)*, suggesting the establishment of a compensatory mechanism, that has been reversed upon treatment with MSI-1436 at 1 μM *(p < 0*.*05)*.

## 4 Discussion

Non-alcoholic fatty liver disease (NAFLD) is a common chronic liver pathology that strongly correlates to obesity and metabolic syndrome that are both characterized by insulin resistance of peripheral tissues [40,41]. In the context of insulin resistance development, endoplasmic reticular (ER) stress is an important factor that hampers the delivery of newly synthesized insulin receptors to the cell surface [42]. ER stress occurs when the amount of unfolded proteins overwhelms the capacities of the ER chaperones and ER-associated degradation (ERAD), this process is triggered by the activation of the expression of unfolded protein response (UPR) chaperones and ERAD [43]. PTP1B is expressed in most of the cell types, and it functions as a negative regulator of insulin signalling. PTP1B knockout mice were protected from diet induced obesity and insulin resistance [44]. PTP1B is also shown to increase in various liver diseases including NASH and ALD [45,46]. Liver specific PTP1B deletion also shown to be protected from hepatic steatosis and ER stress [47]. Thus, several studies were conducted to study the role of PTP1B and their inhibitors in the progression of metabolic disorders. MSI-1436 is a pharmacological inhibitor of PTP1B and shown to increase insulin sensitivity [48,49]. Moreover, MSI-1436 shown to be protected from hepatic steatosis development and lipotoxicity induced ER stress [15,50]. Considering the potential beneficial effects of MSI1436, we decided to analyze the role of MSI-1436 on TM induced ER stress and apoptosis. Here in the current study, we studied the effect of MSI-1436 on ER stress induced hepatocyte apoptosis using HepG2 cell lines as an in-vitro model. We first performed cytotoxicity assay using different concentrations of MSI-1436 on HepG2 cells survival and determined that 1uM and 2uM concentrations of MSI-1436 have shown no cellular, we subsequently utilized these concentrations in the current experimentation. We have treated HepG2 cells with MSI1436 for 1hr followed by combined exposure of TM (5uM) with MSI-1436 for 24hrs. Our results demonstrated that, MSI-1436 attenuated the TM induced apoptosis in HepG2 cells (Fig 1). Pre-treatment with MSI-1436 resulted in increased live cells and reduced depolarized cells after TM induced ER stress (Fig 1). We further analyzed the gene expression of apoptosis mediators p21, p65, Casp-3,6,9 and shown that MSI-1436 can protect HepG2 cells from TM induced cell death by reducing apoptosis (Fig 1). Hepatocyte apoptosis is involved in the progression of NASH to HCC development. Reducing hepatocyte apoptosis in NASH can alleviate development of HCC. Our results indicated that, MSI1436 treatment can suppress ER stress induced hepatocyte apoptosis and thus can be utilized to prevent the progression of NAFLD and subsequent development of NASH and HCC.

In order to understand the implications of MSI-1436 on TM induced ER stress, we have analyzed the markers of ER stress and unfolded protein response (UPR) genes. Our results have demonstrated that MSI-1436 reduced the TM induced ER stress in HepG2 cell lines evident by the reduced gene expression of *Hspa5(BiP)* (Fig 2). Detailed analysis of the three UPR arms suggested that MSI-1436 treatment reduced IRE1a and ATF6 signalling in a dose dependent manner (Fig 2). IRE1, PERK, and ATF6 are the three main ER stress sensors found in mammalian cells that activate UPR signaling cascades in response to ER stress in order to reduce the load of unfolded proteins. IRE1 is also a transmembrane RNase involved in the splicing of X-box-binding protein 1 (XBP1) mRNA, which is a pivotal regulator of the UPR, regulating ER stress adaption. In cells, XBP1 is present in two isoforms: spliced (s) and unspliced (u). XBP1s exhibit transcriptional activity that controls the transcription of UPR-related genes, while XBP1u is a dormant variant of XBP1 that has no transcriptional activity in its unspliced form [51]. Our obtained data demonstrated that MSI-1436 reduced considerably the XBP1 splicing upon exposure of cells to TM, indicating that this PTP1B inhibitor may modulate ER stress and UPR responses via the inactivation of XBP1s transcriptional activity.

Mitochondrial health play’s major role in the progression of NAFLD and the development of NASH and HCC. Hepatic steatosis can lead to development of insulin resistance and ER stress, which can cause mitochondrial dysfunction [14,15]. Damaged mitochondria have to be cleared by the mitophagy [52]. Several reports indicate that, NASH liver is associated with reduced mitophagy, which results in accumulation of damaged mitochondria, releasing the damage associated molecular patterns (DAMPs) [53–59]. DAMPs can initiate the apoptotic signalling leading to hepatocyte death. Nevertheless, increased ROS can result in DNA damage and mutations, which helps some cells to escape from apoptosis pathways, which can initiate the HCC development. In order to avoid these consequences, we are in need for a therapeutic agent which can reduce hepatic apoptosis by reducing mitochondrial damage and prevent mitophagy. In this report, we have found that MSI-1436 treatment to HepG2 cells reduced both mitochondrial depolarization (Fig 3), and ROS accumulation (Fig 4). TM increased mitochondrial fission, which may be a consequence of ER stress induced mitochondrial damage; mito-fission could be an integral part of damaged mitochondrial scavenging by initiating mitophagy. Interestingly, MSI-1436 has been found to reduce mitochondrial fission and increase mitochondrial fusion, indicating that MSI-1436 treatment reduced ER stress induced mitochondrial damage and improved metabolic health (Fig 3). Further, we have also demonstrated that, MSI-1436 treatment significantly reduced Parkin gene expression, which also evidences that MSI-1436 reduced mitochondrial damage, and mitophagy (Fig 3). MSI-1436 has been shown to increase the antioxidants Sod1,2 and catalase which could contribute to the reduced ROS accumulation in HepG2 cells in addition to the improved mitochondrial homeostasis (Fig 4).

In conclusion, using HepG2 cells as an in-vitro model, we have demonstrated that MSI-1436 can be utilized to protect hepatocytes from ER stress and associated mitochondrial dysfunction. Our results underscore the importance of PTP1B inhibitor, MSI-1436 to be used as a therapeutic intervention in reducing hepatocyte apoptosis.

## Supporting information

S1 Table Table_Raw_RT-qPCR Cq Data_ER stress markers: Table summarizing the individual Cq values obtained after RT-qPCR analysis for the key ER stress associated markers. SYBR™ Green dye–based PCR amplification and detection. Data of six technical repetitions for each experimental group. HE: Healthy untreated HepG2 cells; ERS: Endoplasmic reticulum-stressed cells; ERS+MSI: Groups pre-treated with MSI-1436 compound before inducing ERS.
(PDF)

S1 Fig Fig_Raw_Gels images for XBP1 splicing analysis: Original uncropped gel images for XBP1 splicing obtained from agarose gel electrophoresis analysis. Un-spliced XBP1: 281bp, spliced: 255bp. Bands were quantified using Image Studio Lite software (LI-COR Biosciences, USA). HE: Healthy untreated HepG2 cells; ERS: Endoplasmic reticulum-stressed cells; ERS+MSI: Groups pre-treated with MSI-1436 compound before inducing ERS.
(PDF)
