# Supplementary material for: The PTP1B selective inhibitor MSI-1436 mitigates Tunicamycin-induced ER stress in human hepatocarcinoma cell line through XBP1 splicing modulation
Source: PLoS One. 2023 Jan 17;18(1):e0278566. doi: 10.1371/journal.pone.0278566 (PMC9844924; doi:10.1371/journal.pone.0278566)
Supplement: S1 Table — SYBR™ Green dye–based PCR amplification and detection. Data of six technical repetitions for each experimental group. HE: Healthy untreated HepG2 cells; ERS: Endoplasmic reticulum-stressed cells; ERS+MSI: Groups pre-treated with MSI-1436 compound before inducing ERS. (PDF) [file pone.0278566.s001.pdf]

Quantification cycle (Cq) raw data from ER stress markers expression profiling.

Experimental Group	ATF6	BiP	PERK	CHOP	IRE1
HE	26,80322	22,03166	35,06015	26,25811	32,5714
	24,06901	23,4653	32,59544	26,36601	29,20771
	24,66033	22,65057	33,79851	27,38515	30,7427
	27,31957	22,34729	35,5378	25.95296	28,91381
	24,05181	23,31026	33,01081	27,36058	29,52365
	25,01725	22,33498	34,97244	26,55805	30.08442
ERS	24,01393	20,12365	34,19748	24,80461	29,64121
	25,75577	21,60456	35,53725	26,51417	31,69258
	24,02304	20,19701	34,40082	24,67366	30,02605
	24,17555	20,32121	34,79713	24.36951	30.45805
	25,97079	21,15251	34,79093	24.63284	30,96039
	24,05133	20,62129	34,23777	25,20352	29,66324
ERS+MSI-1436_1µM	24,09354	20,00989	34,15039	25,03954	29,39743
	23,58556	20,1406	33,6096	24,81968	30,11251
	23,85149	20,25681	34,59415	24,74159	29,92521
	23,97118	20,32365	34,6789	24.36985	29,31976
	23,61963	20,37051	34,92958	24,0035	30,12259
	24,05805	19,84520	35,35494	25.10258	30,09586
ERS+MSI-1436_2µM	25,24264	20,73339	35,02958	26,80877	29,17514
	24,07394	20,65867	34,3437	25,11066	30,56862
	24,64265	22,76281	35,93366	25,35796	30,73376
	25,40962	20,55214	34,88198	25,30131	30,11582
	24,27921	22,32464	35,33595	24,85231	29,13658
	24,82268	20,63541	34,59371	25,17648	29,55236

Raw Cq data of SYBR™ Green dye–based PCR amplification and detection of reticulum endoplasmic stress associated markers from six technical repetitions for each experimental group.
