# Supplementary material for: The PTP1B selective inhibitor MSI-1436 mitigates Tunicamycin-induced ER stress in human hepatocarcinoma cell line through XBP1 splicing modulation
Source: PLoS One. 2023 Jan 17;18(1):e0278566. doi: 10.1371/journal.pone.0278566 (PMC9844924; doi:10.1371/journal.pone.0278566)
Supplement: S1 Fig — Un-spliced XBP1: 281bp, spliced: 255bp. Bands were quantified using Image Studio Lite software (LI-COR Biosciences, USA). HE: Healthy untreated HepG2 cells; ERS: Endoplasmic reticulum-stressed cells; ERS+MSI: Groups pre-treated with MSI-1436 compound before inducing ERS. (PDF) [file pone.0278566.s002.pdf]

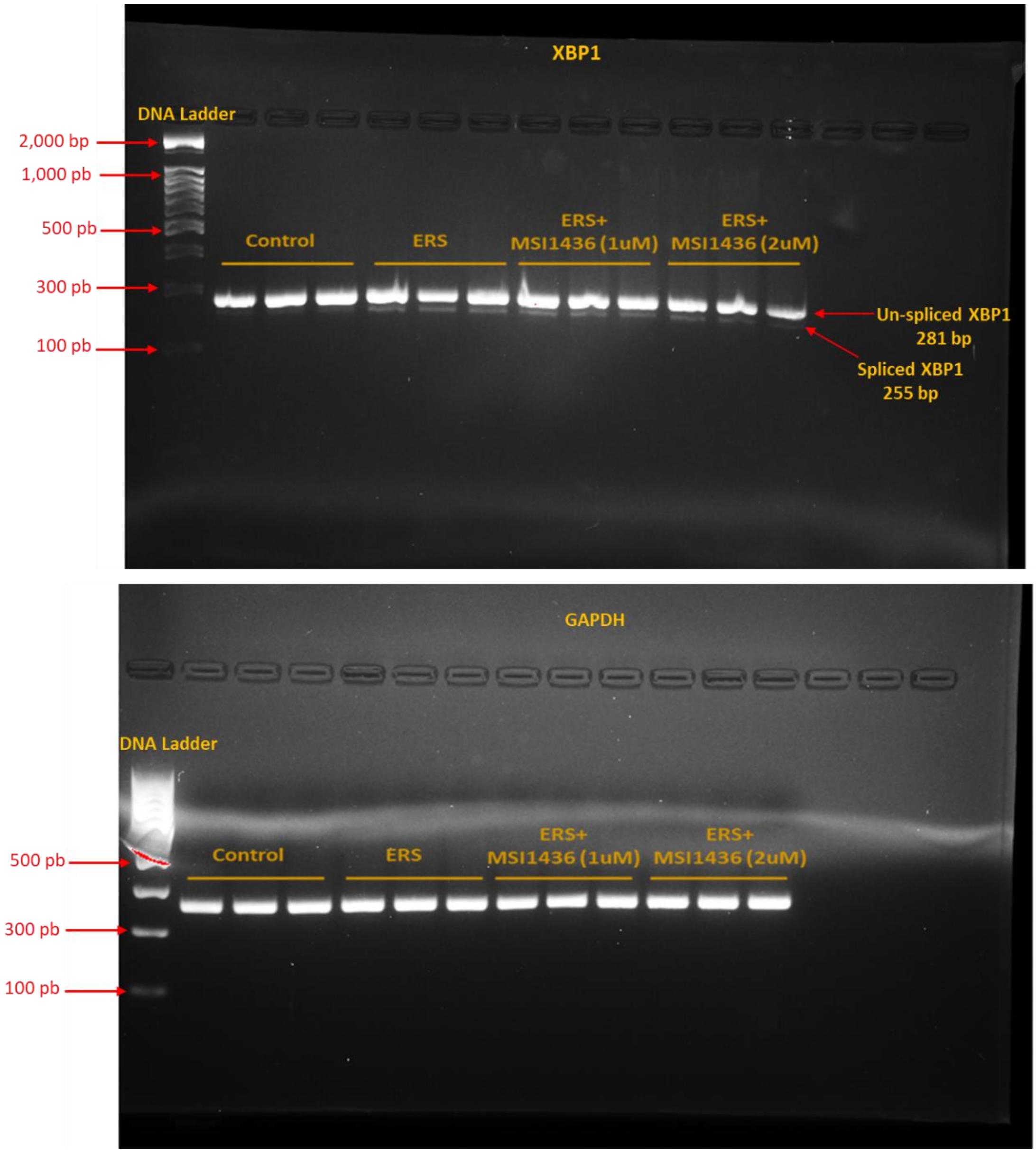

XBP1 amplification product subjected to a 2% agarose gel electrophoresis to visualize the spliced and unspliced XBP1 sequences (un-spliced XBP1: 281bp, spliced: 255bp). Bands were quantified using Image Studio Lite software (LI-COR Biosciences, USA).
